# Potential of Microbial Communities to Perform Dehalogenation Processes in Natural and Anthropogenically Modified Environments—A Metagenomic Study

**DOI:** 10.3390/microorganisms11071702

**Published:** 2023-06-29

**Authors:** Pola Łomża, Tomasz Krucoń, Agnieszka Tabernacka

**Affiliations:** 1 Department of Biology, Faculty of Building Services, Hydro and Environmental Engineering, Warsaw University of Technology, 20 Nowowiejska Street, 00-653 Warsaw, Poland; agnieszka.tabernacka@pw.edu.pl; 2 Department of Environmental Microbiology and Biotechnology, Faculty of Biology, University of Warsaw, 1 Miecznikowa Street, 02-089 Warsaw, Poland

**Keywords:** dehalogenation, organohalogens, halogenated organic compounds, pesticides, metagenome, microbial communities

## Abstract

Halogenated organic compounds (HOCs) pose a serious problem for the environment. Many are highly toxic and accumulate both in soil and in organisms. Their biological transformation takes place by dehalogenation, in which the halogen substituents are detached from the carbon in the organic compound by enzymes produced by microorganisms. This increases the compounds’ water solubility and bioavailability, reduces toxicity, and allows the resulting compound to become more susceptible to biodegradation. The microbial halogen cycle in soil is an important part of global dehalogenation processes. The aim of the study was to examine the potential of microbial communities inhabiting natural and anthropogenically modified environments to carry out the dehalogenation process. The potential of microorganisms was assessed by analyzing the metagenomes from a natural environment (forest soils) and from environments subjected to anthropopression (agricultural soil and sludge from wastewater treatment plants). Thirteen genes encoding enzymes with dehalogenase activity were identified in the metagenomes of both environments, among which, 2-haloacid dehalogenase and catechol 2,3-dioxygenase were the most abundant genes. Comparative analysis, based on comparing taxonomy, identified genes, total halogens content and content of DDT derivatives, demonstrated the ability of microorganisms to transform HOCs in both environments, indicating the presence of these compounds in the environment for a long period of time and the adaptive need to develop mechanisms for their detoxification. Metagenome analyses and comparative analyses indicate the genetic potential of microorganisms of both environments to carry out dehalogenation processes, including dehalogenation of anthropogenic HOCs.

## 1. Introduction

Halogenated organic compounds (HOCs) are one of the major groups of environmental chemicals. HOCs can be formed through biogenic or geogenic processes occurring in nature or through anthropogenic activity. However, the widespread use of HOCs in agriculture and other industries results in their release into the environment and their spread in soil and aquatic environments.

Organohalogens are used in the production of pesticides, pharmaceuticals, solvents, hydraulic and heat transfer fluids, plastics, paints, adhesives, and many other products. The unique physicochemical characteristics of halogens contributes to their synthesis and application on a mass scale. All organohalogens have one or more halogen substituents (F, Cl, Br or I). Halogens are strongly negative and the strength of the carbon–halogen bond changes depending on the halogen. The carbon–fluorine bond is very strong and stable with high polarity. With increasing molecular weight of the halogen, carbon–halogen bond energies decrease in the following order: F > Cl > Br > I [1]. This is a very important feature that affects the metabolism of organohalogens. The halogen substituent may change the compound to which it is attached, increasing the toxicity of the molecule [2,3].

Due to worldwide use of HOCs, all components of the environment (water, soil, and atmosphere) are contaminated by different organohalogens coming from anthropogenic sources (mostly industry). The actual contamination levels of HOCs in air, soil and water in different countries depend on the historical background, industry, legislation prohibiting or authorizing their use, and the fate of HOCs in the environment [4,5,6]. The Stockholm Convention (as of 2021) lists 35 persistent organic pollutants (POPs); all these compounds are organohalogens that are predominantly produced for industrial and agriculture use, with 18 being pesticides. POPs are hazardous chemicals resistant to degradation and transformation; they accumulate in the environment and in fatty tissue, and have high toxicity for many organisms including human beings (Stockholm Convention). These pesticides affect the whole environment, and can be present in soil, groundwater, surface water, and the atmosphere through flow, leaching, and evaporation [7], contaminating many ecosystems.

Some microorganisms may be involved in dehalogenation processes (which remove halogens from organic compounds) [2,8,9,10,11,12]. Studies have identified several metabolic pathways associated with these processes and the enzymes involved in them [13,14,15,16,17]. Dehalogenases, such as haloalkane dehalogenase, haloacetate dehalogenase, 2-haloacid dehalogenase, and dichloromethane dehalogenase, are enzymes that catalyze the breakdown of the carbon–halogen bond. Biological dehalogenation can occur via a metabolic or cometabolic mechanism. In a metabolic mechanism, enzymes produced by microorganisms usually catalyze dehalogenation reactions of specific HOCs. In a cometabolic mechanism, an organohalogen is converted by an enzyme or cofactor produced during microbial metabolism of other compounds. Although organohalides of natural origin are usually biodegradable, anthropogenic organohalides are usually much more resistant to biodegradation.

Our research studied dehalogenation processes in the natural environment and in environments subjected to anthropopressure. The aim of the study was to examine the contribution of microbial communities inhabiting natural and anthropogenically modified environments in dehalogenation processes. Soil samples from the natural environment came from the forested area of Kolonia-Terebiń (near Zamość in southeastern Poland). Samples from anthropogenically modified environments were obtained from a wastewater treatment plant and from agricultural fields where wheat, corn, and cabbage were grown using several halogenated organic pesticides (Ożarów Mazowiecki and Stare Babice, near western Warsaw, Poland).

The potential of microorganisms to transform and thereby remove HOCs from the environment by dehalogenation was analyzed by detecting genes encoding metabolic and cometabolic dehalogenases in the metagenomes of the microbial communities inhabiting the studied soil and sewage environments. Further chemical analysis of the soil samples was undertaken to confirm the presence of halogenated organic compounds in the soil. The study was preceded by an analysis of the taxonomic composition of the microbial communities. The results of our research indicated the presence of several genes encoding enzymes involved in dehalogenation processes in the metagenomes of the microbial communities that inhabit the natural and anthropogenically modified environments being studied. This strongly indicates a role of microbes in the dehalogenation processes in the environment, and indirectly the prevalence of microbial dehalogenation processes.

## 2. Materials and Methods

### 2.1. Site and Sample Description

Investigations were conducted by taking samples from six different sites. Samples S1, S2, S3, and S6 were taken from anthropogenically modified environments in which HOCs were present. Samples S1, S2, and S3 came from agricultural land west of Warsaw, Poland (at Ożarów Mazowiecki, geographical coordinates of sampling sites: 52.230, 20.801; Stare Babice, geographical coordinates of sampling sites: 52.244, 20.857) on which pesticides containing HOCs had been applied—S1 from a field in which maize was cultivated; S2 in a field used to grow cabbages; S3 in a field used to grow wheat; and sample S6 came from a wastewater treatment plant at Stare Babice (west of Warsaw, Poland, geographical coordinates: 52.254, 20.832), from which activated sludge samples were taken. Samples S4 and S5 came from Kolonia-Terebiń (50.705, 23.850, near Zamość in southeastern Poland) and were taken from soil in a natural, non-contaminated forest environment. Soil samples were taken at a depth of 0–30 cm. Ten samples were collected from each site and stored at –80 °C (for DNA extraction) and –20 °C (for chemical analyses).

### 2.2. Physicochemical Analyses of Ground Samples

The pH of the soil was determined by suspension of soil in 1 mol/L potassium chloride solution (pH in KCl) according to EN ISO 10390: 2022. To determine the soil’s organic matter content, weighed amounts of soil were initially dried at 105 °C until dry, and then calcinated at 550 °C for three hours and reweighed. The heavy metal content was determined using inductively coupled plasma optical emission spectrometry (ICP-OES). Halogen (F, Cl, Br, I) content was determined using ion chromatography. The heavy metal and halogen content were measured at the National Institute of Geology of the Polish Academy of Sciences in Warsaw, Poland.

### 2.3. Extraction and Gas Chromatography Analyses

The analytical procedure of extraction was conducted according to the standard ISO 10382: 2002. Soil samples (10 g, grain size ≤ 0.20 mm) were extracted in a Soxhlet extractor with a solvent mixture of hexane/acetone (70:30 *v*/*v*). Soil extracts were concentrated, and the solvent was exchanged to hexane. The concentrated extracts were passed through a glass column packed with glass wool, 2 g of deactivated aluminum oxide (15% in Milli-Q water), and 1 cm of anhydrous sodium sulfate; then it was eluted with 20 mL of petroleum ether at 40–60 °C. The solvent was exchanged to hexane following vacuum evaporation, and the extracts were concentrated to 1 mL. A prepared solution (of 1 µL) was introduced into the GC injection port via 7683B ALS (automatic liquid sampler). Gas chromatography analysis was performed using an Agilent Technologies gas chromatograph GC 7890A coupled with a mass spectrometer (MS 5975C VL MSD). The GC/MS was equipped with a DB-5 column (30 m × 0.25 mm × 0.25 µm) and operated with an injector temperature of 280 °C and a AUX line temperature of 280 °C. The initial oven temperature of 70 °C was held for 2 min, and then increased to 150 °C at a rate of 8 °C/min, then increased to 200 °C at a rate of 3 °C/min, then increased to 280 °C at a rate of 8 °C/min and held at the final temperature for 10 min. Helium (1 mL/min) was used as a carrier gas. The MS quad and MS source temperatures were held at 150 °C and 230 °C, respectively. In addition, the content of organochlorine pesticides in soil and sludge samples was obtained with a gas chromatograph coupled with electron capture (GC-ECD) (performed by ALS Laboratory Poland). The GC-ECD was equipped with a DB-5 column (30 m × 0.25 mm × 0.25 µm) and operated with an injector temperature of 280 °C and ECD detector temperature of 310 °C. The temperature program was the same as for the GC/MS analyses. Helium (1 mL/min) was used as a carrier gas and nitrogen (40 mL/min) was used for the ECD detector.

### 2.4. Isolation of DNA

The metagenomic DNA was extracted from 0.5 g of soil samples using a Soil DNA Mini Kit (Syngen) according to the manufacturer’s protocol. Extracted DNA was further purified from humic acid contamination using the Environmental DNA & RNA Purification Kit (EURx) according to the manufacturer’s protocol. Ten samples from each site (S1–S6) were subjected to the DNA extraction protocol described above. The quality of the extracted and purified DNA was checked using a spectrophotometer to measure the A260/280 ratio (to check samples for RNA contamination) and the A260/230 ratio (to check samples for humic acid contamination), and PCR was performed to detect 16S rDNA for all samples. The A260/280 ratio in the non-contaminated sample was in the range of 1.8–2.0 and the A260/A230 ratio was in the range of 2.0–2.2. All 60 samples underwent the quality control tests. After the quality control tests, the 10 DNA samples from each site were mixed into single sample. The resulting six samples were labeled S1, S2, S3, S4, S5, and S6, respectively. These six samples were then sequenced.

### 2.5. Metagenome Sequencing and Analysis

Genomic DNA concentration was measured prior to the library preparation procedure by fluorimetry using the PicoGreen reagent (Life Technologies Thermo Scientific™, ABO sp. z o.o., Gdańsk, Poland). The measurement was performed using the Tecan Infinite apparatus. Library preparation for the six DNA samples (from sites S1–S6) was undertaken as follows. Metagenomic DNA was fragmented by sonication on a Covaris E210 device (Covaris Ltd, Brighton, UK) in accordance with the parameters recommended for library preparation for sequencing using Illumina technology. Libraries were prepared using the NEBNext Ultra II DNA Library Prep Kit for Illumina (Version 7.0_9/22, New England Biolabs, Ipswitch, MA, USA) according to the manufacturer’s instructions. Each sample was uniquely marked with TruSeq UD indices. Sequencing was performed using the NovaSeq6000 sequencer, paired-end (PE) technology, 2 × 150 nt, using NovaSeq 6000 S4 Reagent Kit v1.5 300 cycles (Illumina) reagents according to the manufacturer’s protocol. The readings were filtered with Cutadapt version 3.0. Quality control of the sequencing results was performed using the FastQC program. Subsequent data analysis was performed using the Trimmomatic tool (v. 0.38) [18] (SLIDINGWINDOW:4:20, HEADCROP:5, and MINLEN:100). Human reads were removed using BMTagger (v. 1.1.0) [19] and the human genome database GRCh38/hg38. The obtained unassembled reads were assigned to the taxa using Kraken2 (v. 2.0.8-beta) [20] and the prokaryotic, fungal, and viral protein sequences included in the NCBI RefSeq database [21]. Metagenomes were assembled using SPAdes v3.13.1 [22] and the trimmed reads were mapped to the contigs using Bowtie2 version 2.3.5.1 [23]. Contigs shorter than 1 kb or with an average coverage of less than five were discarded from the assembly. QUAST (v. 5.0.2) [24] was used to evaluate the assembled contigs. Annotation was then performed using PROKKA (v. 1.13) [25] with the default parameters and using KofamScan (v. 1.3.0) with KOfam HMM profiles (v. 26.04.2021) [26]. The hits with a bit score < 60 and e-value > 1 × 10^−5^ were discarded. Based on KO numbers and KEGG ortholog classification, genes were assigned to metabolic pathways. Duplicates were removed using MarkDuplicates from the Picard toolkit [27]. For all samples, coverage was expressed as transcripts per million (tpm) according to Equations (1) and (2) [28]. The taxonomic assignment of the sequences was conducted using the last common ancestor method with default parameters (taxator-tk v1.3.3e) [29].

(1) $$tpm=\frac{r_{g}\times rl\times 10^{6}}{fl_{g}\times t}$$

(2) $$t=\underset{g\epsilon G}{\sum }\frac{r_{g}\times rl}{fl_{g}}$$

where:

*tpm*: abundance of the genes expressed as transcripts per million;

*r_g_*: reads mapped to gene g;

*rl*: read length;

*fl_g_*: feature length;

*t:* the total number of reads sampled in a sequencing run.

## 3. Results

### 3.1. Physical and Chemical Analyses of Studied Samples

All soil samples were slightly acidic (6.2–6.4 pH). The average content of organic matter in the samples was as follows: S1—3.44%, S2—3.90%, S3—2.64%, s4—4.57%, S5—14.21%, and S6—75.58%. Heavy metal content was low in all analyzed samples (Table 1), as measured using inductively coupled plasma optical emission spectrometry (ICP-OES) (assays were performed by ALS laboratory Poland). The halogen content in the soil samples was obtained using ion chromatography (assays were performed by the Polish Geological Institute); the results are presented in Table 2.

Several HOCs were identified using gas chromatography coupled with mass spectrometry (GC-MS), of which the most interesting were DDT (dichlorodiphenyltrichloroethane), DDE (dichlorodiphenyldichloroethylene) and esters of trichloroacetic acid (in samples S1–S3), 2-chloropropanoic acid octadecyl ester, chlorhexadecane, trifluoroacetoxytetradecane, tridecyl heptafluorobutyric acid, 3,6-dichloro-9H-carbazole, 1-chlorooktadecane, 1-idodmetyloundecane, and esters of trichloroacetic acid (in samples S4–S5). It should be mentioned that HOCs were present in trace amounts in all the tested soil samples. Samples from natural environments (S4 and S5) were characterized by a greater variety of HOCs than those found in the samples from anthropogenically modified locations. In addition, the content of organochlorine pesticides in soil and sludge samples, which was measured with a gas chromatograph coupled with electron capture (GC-ECD), was also examined. Chlorinated pesticides that were present in samples in higher than trace amounts were isomers DDD (dichlorodiphenyldichloroethane), DDT, and DDE; their average concentrations are presented in Table 3. The value of 4,4′-DDE in the S2 sample exceeds the Polish legal requirements for content in agricultural land (Ordinance of Minister of Environment 2016, Law Journal 2016, position 1395). Moreover, only sample S2 (soil) and sample S6 (activated sludge) contained DDD, DDT, and DDE isomers in concentrations higher than the trace amounts. Supplementary Table S2 contains a statement of the average content of all analyzed organochlorine pesticides that were present in trace amounts in the soil and sludge samples.

### 3.2. General Characteristics of Metagenomes of Natural and Anthropogenically Modified Environments

Metagenomic sequencing of the samples from the studied environments was performed using Illumina’s next-generation sequencing system. This resulted in an average of 47,168,834.5 raw pairs of reads with a length of 150 bp. After quality control (filtering of low quality reads, impurities, and chimeras), on average, a total of 47,080,794 high-quality pair reads remained, which had an average length of 143.76 ± 0.16 bp. Taxonomic classification was possible for 51.48 ± 3.36% of the metagenomic reads, while functional annotation was possible for 58 ± 9.69% of the reads. The results of the metagenomic DNA sequencing are deposited at https://www.ncbi.nlm.nih.gov/bioproject/PRJNA889689, accessed on 12 October 2022 (sample names: S1–S6).

### 3.3. Taxonomic Composition and Diversity of Microbial Communities

**Figure 1:**
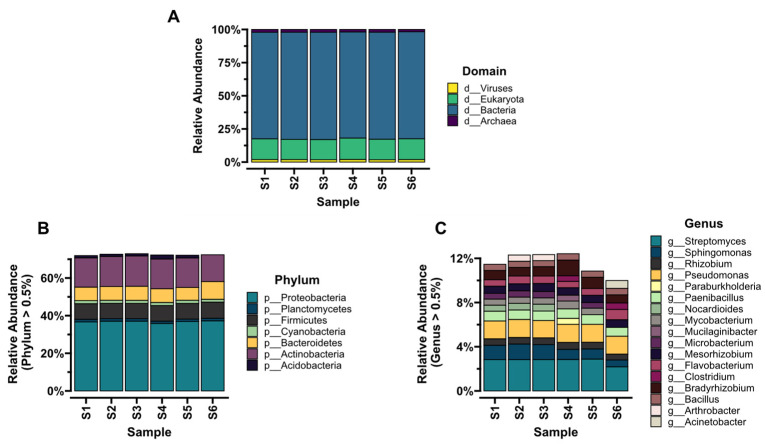
Taxonomic diversity of the metagenomes of the natural and anthropogenically modified environments in percent: domain (**A**), dominant (relative abundance > 0.5%) phyla (**B**), dominant (relative abundance > 0.5%) genera (**C**).

In the metagenomes of soil environments, approximately 87% of the assigned reads were related to bacteria. In all the sites studied, the dominant phylum was *Proteobacteria*, accounting for 32% (S4) to 43% (S6) of the assigned reads, followed by *Actinobacteria* for S1, S2, S3, and S6 with 10–12% of the assigned reads, and *Acidobacteria* for S4 and S5 with 11–18% of the assigned reads. The dominant classes in S1–S5 were *Alphaproteobacteria*, ranging from 13 to 16% of the assigned reads, and *Actinomycetia*, with 9–11% of the assigned reads, while the dominant class in S6 was Betaproteobacteria with 16% of the assigned reads. In the case of both the agricultural and non-agricultural environments (S1–S5) and sewage treatment plant (S6), *Hyphomicrobiales* and *Burkholderiales* were the dominant orders. The dominant families were *Sphingomonadaceae* in S1–S3, *Nitrobacteraceae* in S4 and S5, and *Nitrospiraceae* in S6. The most numerous genera were *Sphingomonas*, *Streptomyces*, *Pseudomonas*, and *Bradyrhizobium* (S1–S5), and *Nitrospira*, *Propionivibrio*, *Pseudomonas*, and *Acinetobacter* in S6 (Figure 1).

### 3.4. Occurrence of Dehalogenase Genes in Natural and Anthropogenically Modified Environments

**Figure 2:**
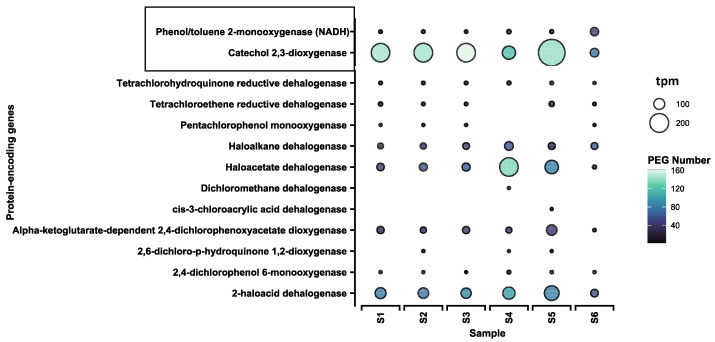
Relative abundance of the genes (expressed in transcripts per million (tpm) reads) of metabolic and cometabolic dehalogenases identified in the metagenomes from the natural and anthropogenically modified environments. The frame indicates the cometabolic dehalogenases. PEG—protein-encoding genes.

We found 11 metabolic and 2 cometabolic genes encoding dehalogenases, with a relative abundance of 1867 and 1214 transcripts per million (tpm), respectively (Figure 2). The greatest abundance of metabolic dehalogenase genes was found in S5 (475 tpm) and in S4 (450 tpm), with the lowest in S6 (136 tpm); the same pattern was observed in the case of cometabolic dehalogenase genes—the highest abundance of cometabolic dehalogenase genes was found in S5 (312 tpm) and the lowest in S6 (134 tpm).

The most abundant metabolic dehalogenase gene was 2-haloacid dehalogenase, with a relative abundance of 611 tpm, and the sample with the highest 2-haloacid dehalogenase gene relative abundance was S5 (149 tpm). Another highly abundant dehalogenase gene was haloacetate dehalogenase (524 tpm), with the highest relative abundance in S4 (198 tpm) and S5 (133 tpm), followed by haloalkane dehalogenase (292 tpm) and alpha-ketoglutarate-dependent 2,4-dichlorophenoxyacetate dioxygenase (286 tpm); again, the samples with the highest relative abundances were S4 and S5, respectively. The most abundant cometabolic dehalogenase gene was catechol 2,3-dioxygenase, with a relative abundance of 1092 tpm, greatest in S5 (300 tpm), while lowest in S6 (69 tpm) (Figure 2). The phenol/toluene 2-monooxygenase gene was better represented in the anthropogenically modified site (S6) than in other environments. Regarding other gene encoding metabolic dehalogenase of note, pentachlorophenol monooxygenase was not observed in samples from natural environments, and dichloromethane dehalogenase and *cis*-3-chloroacrylic acid dehalogenase were only observed in the samples from natural environments (S4 and S5) (Figure 2). The accession numbers of all the detected dehalogenases are presented in Supplementary Table S1.

**Figure 3:**
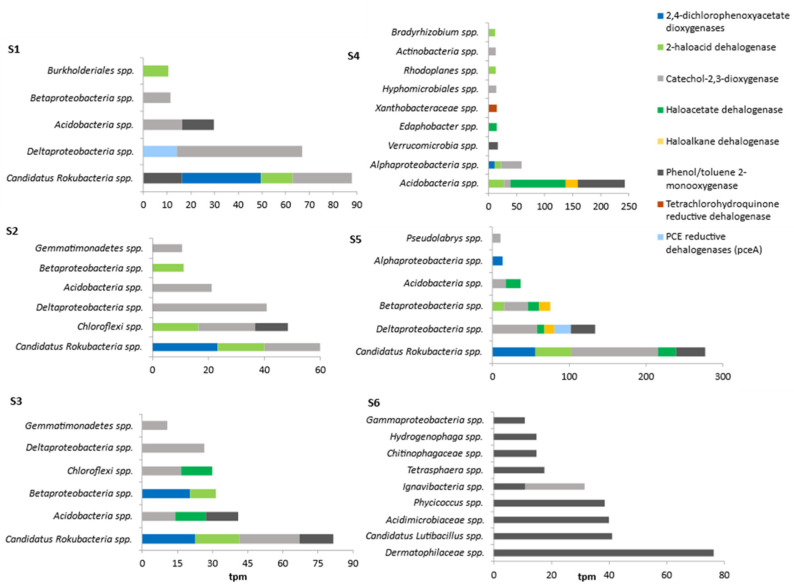
Relative abundance of the genes (expressed in transcripts per million (tpm) reads at the taxon level) encoding metabolic and cometabolic dehalogenases in the metagenomes from the natural and anthropogenically modified environments.

The taxa to which the most abundant dehalogenase genes were assigned included *Candidatus Rokubacteria* spp. (five dehalogenase genes were assigned to this taxon: 2,4-dichlorophenoxyacetate dioxygenases, 2-haloacid dehalogenase, haloacetate dehalogenase, phenol/toluene 2-monooxygenase, catechol-2,3-dioxygenase, with the total reads over 500 tpm) and *Acidobacteria* spp. (five dehalogenase genes were assigned: 2-haloacid dehalogenase, haloacetate dehalogenase, haloalkane dehalogenase, phenol/toluene 2-monooxygenase, catechol-2,3-dioxygenase, with the total reads over 370 tpm). In addition to these taxa, dehalogenase genes were also assigned to (or had large number of reads in tpm) Delta-, Beta-, and Alpha-*proteobateria* spp. and *Chloroflexi* spp. (Figure 3).

### 3.5. Correlations Analyses

**Figure 4:**
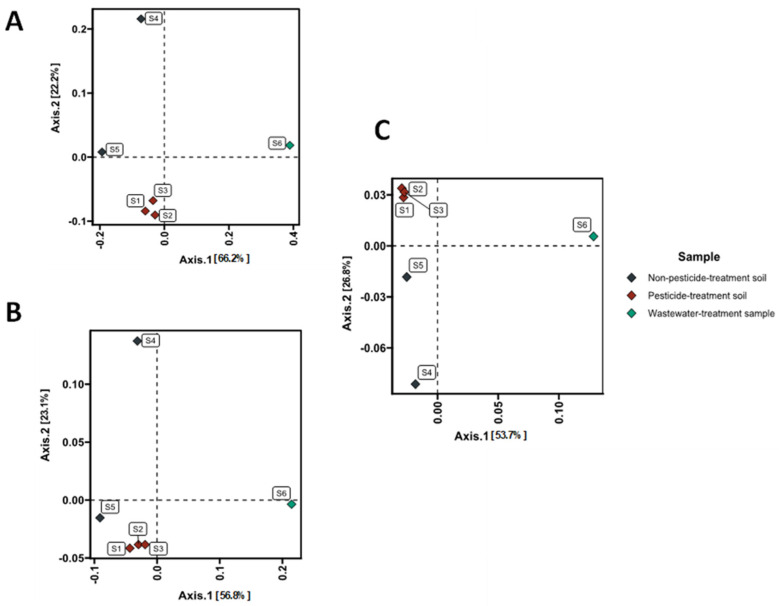
Principal components analysis (PCA) of the similarities between the microbial communities in the metagenomes from the natural and anthropogenically modified environments. PCA of similarity according to (**A**) dehalogenases genes; (**B**) all identified genes; (**C**) taxonomy.

A principal components analysis (PCA) of the similarities between the studied microbial communities based on metagenomes showed high similarities between the microbiota communities from the three different agricultural fields. PCA captured 79–88% of the total variance within the system. It also showed significant differences between the samples from natural environments, despite sampling points came from the same forested area. These distinctions may arise from differences in the soil characteristics; for example, they may be caused by differences in organic matter content (S4—4.57%, S5—14.21%) (Figure 4).

**Figure 5:**
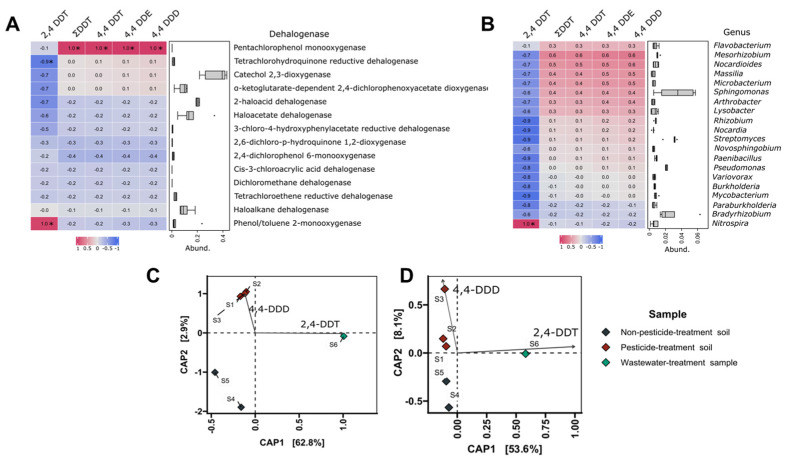
(**A**) Correlation analysis shows relationship between the content of DDT derivatives and genes encoding dehalogenases. Abundance indicates the amount of each dehalogenase gene in the analyzed environments. (**B**) Correlation analysis shows relationship between the content of DDT derivatives and taxonomic composition in analyzed environments at the genus level. Abundance indicates the amount of each genus in analyzed environments. (**C**) Canonical analysis of principal coordinates (CAP) shows relationship between the content of DDT derivatives and presence of proper genes encoding dehalogenases in analyzed environments. (**D**) Canonical analysis of principal coordinates (CAP) shows relationship between the content of DDT derivatives and the taxonomic composition in analyzed environments at the genus level. The length of each vector line is proportional to the strength of the correlation. (*) indicates a statistically significant result (*p*-value < 0.05).

The data indicate that the higher content of 2,4-DDT in the analyzed environments is associated with the increased occurrence of genes encoding phenol/toluene 2-monooxygenase, while the occurrence of pesticides containing chlorine atoms in the *para* position (4,4-DDT, 4,4-DDE, 4,4-DDD) is associated with the occurrence of genes encoding pentachlorophenol monooxygenase (Figure 5A). The correlation analysis presented in Figure 5B shows that the genera *Flavobacterium*, *Mesorhisobium*, *Nocardioides*, *Massilia*, *Microbacterium*, *Sphingomonas*, *Arthrobacter*, and *Lysobacter* are probably tolerant or have detoxification/transformation mechanisms for 4,4-DDT, 4,4-DDE, and 4,4-DDD, while the genus *Nitrospira* is tolerant or has detoxification/transformation mechanisms for 2,4-DDT. Another issue is that canonical analysis of principal coordinates (CAP) (Figure 5C) indicated a higher similarity between samples S1, S2, and S3 based on the content of 4,4-DDD and dehalogenase-encoding genes. The similarity between samples was also confirmed by the correlation based on the content of 4,4-DDD and taxonomy (Figure 5D).

**Figure 6:**
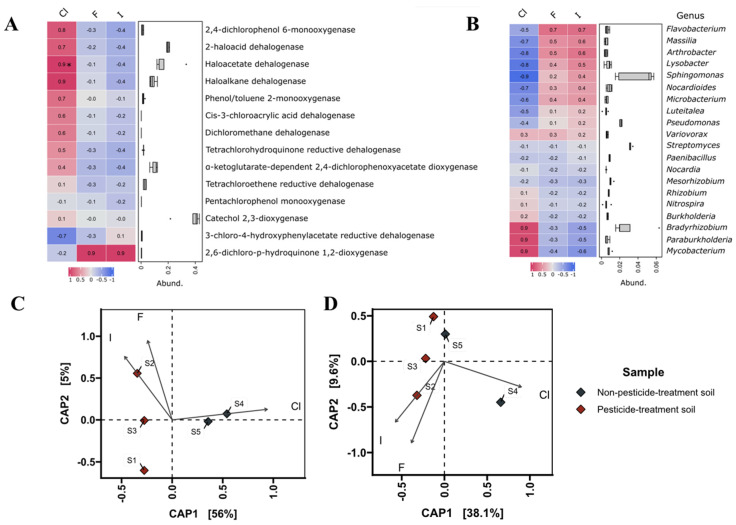
(**A**) Correlation analysis shows relationship between the content of fluorine, chlorine, iodine, and genes encoding dehalogenases. Abundance indicates the amount of each dehalogenase gene in analyzed environments. (**B**) Correlation analysis shows relationship between the content of fluorine, chlorine, iodine, and taxonomic composition in analyzed environments at the genus level. Abundance indicates the amount of each genus in analyzed environments. (**C**) Canonical analysis of principal coordinates (CAP) shows relationship between the content of fluorine, chlorine, iodine, and presence of proper genes encoding dehalogenases in analyzed environments. (**D**) Canonical analysis of principal coordinates (CAP) shows relationship between the content of fluorine, chlorine, iodine, and the taxonomic composition in analyzed environments at the genus level. The length of each vector line is proportional to the strength of the correlation. (*) indicates a statistically significant result (*p*-value < 0.05).

The correlation analysis between the content of fluorine, chlorine, iodine, and the genes encoding dehalogenases shows that there is similarity between samples S1, S2, and S3 and between samples S4 and S5 (Figure 6C), while the analysis of correlation between the content of halogens and the taxonomy indicates a higher similarity between sample S5 and samples S1, S2, and S3, rather than S4 (Figure 6D). In chloride-rich environments, there is an increased presence of the following genes in bacterial genomes: 2,4-dichlorophenol 6-monooxygenase, 2-haloacid dehalogenase, haloacetate dehalogenase, haloalkane dehalogenase, phenol/toluene 2-monooxygenase, *cis*-3-chloroacrylic acid dehalogenase, dichloromethane dehalogenase, tetrachlorohydroquinone reductive dehalogenase, and alpha-ketoglutarate-dependent 2,4-dichlorophenoxyacetate dioxygenase (Figure 6A). Genera such as *Mycobacterium, Paraburkholderia*, and *Bradyrhizobium* may have some preferences for environments with a higher content of chlorides, while genera such as *Flavobacterium*, *Massilia*, *Arthrobacter*, *Lysobacter*, *Sphingomonas*, *Nocardioides*, and *Microbacterium* prefer environments with a higher content of fluoride and iodide (Figure 6B).

## 4. Discussion

### 4.1. The Importance of Microbial Dehalogenation for the Environment

Microbial dehalogenation seems to be a crucial process in halogenated organic pesticide transformation and might result in faster degradation of these toxic chemicals. Knowledge of the influence of different conditions on dehalogenation might be significant in improving the process. With intensive farming activities, many halogenated organic pesticides have been brought to the surface and exposed to a deeper layer of ground and groundwaters and to the biosphere, where microorganisms play a key role in halogen cycling. Exposure to high concentrations of HOCs and frequently changing pesticides delivered to the ground and groundwaters for extended periods selects for microorganisms better adapted to such conditions. Microbes may develop strategies to survive and transform HOCs. Understanding these strategies and their limitations will enable us to better employ microbial dehalogenation mechanisms. In addition to providing a better understanding of the influence of physicochemical conditions on the dehalogenation processes that help in effective bioremediation, metagenomic analysis allows investigation of the metabolic potential of microorganisms to conduct biochemical dehalogenation reactions. Therefore, in this study, we employed integrated surveys such as chemical and metagenomic analyses to provide metabolic information on the microbiota in natural and anthropogenically modified environments. We observed that the metabolic potential of the microbiota demonstrated some similarities in the anthropogenically modified and natural sites.

### 4.2. An Overview of the Studied Environments

One of the basic steps in the characterization of the environment inhabited by the microorganism communities of interest is the assessment of the presence of coexisting pollutants. Therefore, we conducted an analysis of the composition and content of heavy metals, halogens, and the presence of petroleum products in the analyzed soils. In all analyzed soil samples, no increased heavy metal content was found (Table 1). No petroleum hydrocarbons were identified. In the case of fluorine, chlorine, and bromine, their content was consistent with the average concentrations observed in the lithosphere. According to Kirk [30], the concentrations of fluorine, chlorine, bromine, and iodine in the lithosphere are 770, 550, 1.6, and 0.3 (ppm), respectively. Fuge [31] indicated that the fluorine content in the crust of the Earth is 557 ppm; Geilfus [32] notes an average concentration of chlorine in the lithosphere of 480 ppm; and other authors have calculated a bromine content of 2.1 ppm [33] and iodine content of 0.3 ppm [34]. The concentration of iodine in the S2 sample was almost fifty times higher than the observed content of iodine in the lithosphere (Table 2). The reason for such a high concentration is difficult to state unequivocally.

Another important finding is that no halogenated organic pesticides that were in use on the agricultural land before sampling were identified in the analysis of the samples. This could be due to the migration of pesticides to deeper layers of the soil where there are more suitable anaerobic conditions and/or the dispersal of pesticides a long distance from the place of the use. Organohalogens reach groundwater mainly through water that infiltrates the soil and passes through the unsaturated zone below to the water surface. The transport of halogenated organic compounds to groundwater is caused by rainfall or irrigation systems [35]. Organohalide compounds are largely retained by soil and aquifers, and the transport of these compounds into and within groundwater is difficult to predict. Both HOCs and their degradation products can move directly to groundwater through mobile zones, but some compounds are trapped in immobile zones, and they can be gradually released to groundwater by leaching and diffusion, sometimes long after being released into the environment. In addition, any halogenated pesticides used at the sampling sites could have already been transformed and degraded by microorganisms inhabiting the soil environment and entered the halogen cycle.

### 4.3. The Characteristics of Identified Dehalogenases Genes

A majority of the dehalogenase genes detected in both anthropogenically modified environments and natural environments fell under bacterial taxa (Figure 3), primarily *Candidatus Rokubacteria* spp. in anthropogenically modified environments and *Candidatus Rokubacteria* spp. and *Acidobacteria* spp. in natural environments. This indicates that bacteria play a key role in the transformation of HOCs in the environment.

Dehalogenases genes were most abundant in samples from the natural environment (S4 and S5). Apart from phenol/toluene 2-monooxygenase, 2,6-dichloro-*p*-hydroquinone 1,2-dioxygenase, and pentachlorophenol monooxygenase, the remaining ten genes were best represented in samples S4 and S5. This may indicate the evolutionary adaptation of organisms to the transformation of HOCs, which must have been present in the natural environment in amounts that affect microorganisms, forcing organisms to develop mechanisms for their transformation. To date, about 5000 natural HOCs have been discovered [36,37,38,39]. They are found in various aquatic and terrestrial environments and are formed as a result of biological, biogeochemical, and geogenic processes. Biogenic organohalides are produced by bacteria, fungi, sponges, lichens, algae, plants, mollusks, polychaetes, jellyfish, insects, and mammals [37,40,41]. The presence of dichloromethane dehalogenase and *cis*-3-chloroacrylic acid dehalogenase genes only in the samples from natural environments suggests a natural origin for the dichloromethane and vinyl chloride in soils of the forest. Gribble [37] reached a similar conclusion, observing that dichloromethane may have natural sources from biomass combustion or wetlands, and indicated that vinyl chloride can be produced abiotically in soil during the oxidative degradation of soil matter. Naturally produced trichloromethane can be a precursor of dichloromethane in soil samples as well.

Conversely, the absence of pentachlorophenol monooxygenase gene in the metagenomes that came from natural environments and its presence in anthropogenically modified environments suggests that the pentachlorophenol monooxygenase gene is involved in the dehalogenation of xenobiotic halogenated organic compounds.

### 4.4. The Contribution of Microorganisms in HOC Dehalogenation

Bacteria have evolved different strategies to remove the halogen substituent from HOCs. Generally, these include oxygenolytic, hydrolytic, and reductive approaches. Oxygenolytic dehalogenation proceeds strictly in aerobic conditions, in which oxygen is necessary as a cofactor; hydrolytic and reductive dehalogenation may occur in both aerobic and anaerobic conditions, but most often reductive dehalogenation occurs in the strict absence of oxygen [42]. In our study, the metagenome analysis indicated the predominance of oxygenolytic and hydrolytic dehalogenation in the studied environments. In all investigated soil environments, based on the metagenome analysis, targeted metabolic dehalogenation reactions prevailed, while in samples from the sewage treatment plant, metabolic and cometabolic processes were balanced. The main dehalogenase-encoding genes were the genes for 2-haloacid dehalogenase (611 tpm) among the metabolic dehalogenases and catechol 2,3-dioxygenase (1092 tpm) among the cometabolic dehalogenases.

One of the mechanisms that results in the removal of halogen substituents is HCl elimination by dehydrohalogenases, present in organisms including *Sphingomonas* [43]. Substitutive dehalogenation represents a different approach to the removal of the halogen group, involving a variety of metal and metalloid hydrides and HOCs, with one mechanism based on nucleophilic substitution. This type of reaction is also called hydrodehalogenation. According to some research [44,45,46,47], the enzymes involved in this mechanism are s-triazine hydrolase, 2,6-dichlorohydroquinone chlorohydrolase, and atrazine chlorohydrolase, which are present in *Pseudomonas, Rhodococcus,* and *Rhizobium*. Aromatic substitution reactions are carried out by several bacterial strains belonging to the genera identified in the studied metagenomes, including *Pseudomonas, Arthrobacter, Acinetobacter, Nocardia*, and *Corynebacterium*. These reactions are catalyzed by 2-haloacid dehalogenase and 1-chloroalkane halidohydrolase, as well as enzymes encoded by genes present in the studied metagenomes, such as haloalkane dehalogenase and haloacetate dehalogenase (Figure 2).

Within oxidative dehalogenation, cometabolic oxidation presents a very important group of reactions that enable the dehalogenation of alkanes, alkenes, and aromatic compounds. These are catalyzed by multiple enzymes, mainly mono- and dioxygenases (such as toluene 2,3-dioxygenase, alkene monooxygenase, propane monooxygenase, cytochrome P450), as well as those found in the studied metagenomes (catechol 2,3-dioxygenase, phenol/toluene monooxygenase, which are known to degrade of a variety of organic compounds) (Figure 2). The important bacterial genera involved in oxidative dehalogenations are *Pseudomonas, Alcaligenes, Sphingomonas, Burkholderia*, and *Mycobacterium* [41,44,45,46].

Reductive dehalogenation (anaerobic) processes are carried out by genera such as *Dehalobacter, Dehalobacterium*, and *Dehalococcoides* [48,49,50,51,52,53,54]. These were found in all studied metagenomes, but in small relative abundance (<0.01%); however, two other reductive dehalogenases were found, tetrachloroethene reductive dehalogenase and tetrachlorohydroquinone reductive dehalogenase, the latter only identified in the metagenome that came from the wastewater treatment plant. Table 4 lists and describes all the dehalogenase genes identified in natural and anthropogenically modified environments in this study. Of course, the presence of genes detected in the studied metagenomes does not equate to their expression and production of active protein. However, laboratory work conducted on the ability of the microflora of the environments analyzed in this study confirmed the metabolic capacity of these microorganisms to transform select HOCs (data not presented).

## 5. Conclusions

In summary, the identification of microbial genes known to be involved in the halogen cycle in the metagenomes of microbial communities of natural and anthropogenically modified environments provides evidence for the existence of microbial dehalogenation pathways in the studied environments and evolutionary adaptation of microorganisms to dehalogenation. The open question is how can we influence the expression of these genes? It can be assumed that the described dehalogenation processes may be an important element of halogenated organic compound transformation in the environment.

## Figures and Tables

**Table 1:** Content of heavy metals in ground and sediment samples, measured using inductively coupled plasma optical emission spectrometry (ICP-OES).

Sample	As	Ba	Cr	Sn	Zn	Cd	Co	Cu	Mo	Ni	Pb	Fe
	mg/kg d.w.											%
S1	<3	33	10	<2	19	<0.5	3	7	<0.5	7	11	0.71
S2	<3	68	11	<2	39	<0.5	3	22	<0.5	13	14	0.50
S3	4	91	14	2	58	<0.5	3	21	<0.5	9	21	1.08
S4	1.8	44	15	<1	21	<0.5	4	7	<0.5	9	13	0.99
S5	<0.5	76	16	<1	38	<0.5	3	21	<0.5	15	16	0.77
S6	2.6	132	22	12	470	0.8	3	199	4	16	11	1.13

**Table 2:** The total content of halogens (fluorine, chlorine, bromine, and iodine) in soil samples, measured using ion chromatography.

Sample	F	Cl	Br	I
	mg/kg	mg/kg	mg/kg	mg/kg
S1	<5.00	37.5	<5.00	<5.00
S2	31.5	45.5	<5.00	14.5
S3	13.5	78.0	<5.00	<5.00
S4	13.5	127	<5.00	<5.00
S5	13.5	127	<5.00	<5.00

**Table 3:** Organochlorine pesticide content in soil and sediment samples obtained using a gas chromatograph coupled with an electron capture detector (GC-ECD).

Sample	2,4- DDD	2,4- DDT	4,4′- DDD	4,4′- DDE	4,4′- DDT	Sum of 4 Isomers of DDT	Sum of 6 Isomers of DDT
	mg/kg d.w.						
S1	<0.010	<0.010	<0.010	<0.010	<0.010	<0.040	<0.060
S2	<0.010	0.012	0.014	0.171	0.062	0.259	0.259
S3	<0.010	<0.010	<0.010	<0.010	<0.010	<0.040	<0.060
S4	<0.010	<0.010	<0.010	<0.010	<0.010	<0.040	<0.060
S5	<0.010	<0.010	<0.010	<0.010	<0.010	<0.040	<0.060
S6	0.011	0.039	<0.010	0.016	0.014	0.069	0.080

**Table 4:** Characteristics of dehalogenases encoded by genes identified in the metagenomes from the natural and anthropogenically modified environments.

Type of Enzyme	Name of Enzyme	Reaction	Characteristic of Dehalogenation Mechanism	References
**Metabolic dehalogenases**	pentachlorophenol monooxygenase	H^+^ + NADPH + O_2_ + pentachlorophenol = 2,3,5,6-tetrachloro-1,4-benzoquinone + chloride + H_2_O + NADP^+^	Pentachlorophenol monooxygenase belongs to oxidoreductases (monooxygenases) responsible for dechlorination of pentachlorophenol to tetrachlorobenzoquinone.	[55,56]
	2,6-dichloro-*p*-hydroquinone 1,2-dioxygenase	2,6-dichlorohydroquinone + O_2_ + H_2_O = 2-chloromaleylacetate + hydrochloric acid	2,6-dichloro-*p*-hydroquinone 1,2-dioxygenase is an oxidoreductase (dioxygenase) and is responsible for opening the aromatic ring of 2,6-dichloro-*p*-hydroquinone (cleaves the aromatic ring between a hydroxyl group and a chlorine group) to produce 2-chloromaleylacetate.	[57,58]
	2-haloacid dehalogenase	2-haloacid + H_2_O = 2-hydroxyacid + halide	Dehalogenates 2-haloalkanoic acids to the hydroxyalkanoic acids, with inversion of configuration at C-2. Acts on 2-haloalkanoic acids whose carbon chain lengths are five or less.	[46,59]
	2,4-dichlorophenol 6-monooxygenase	2,4-dichlorophenol + H^+^ + NADPH + O_2_ = 3,5-dichlorocatechol + H_2_O + NADP^+^	2,4-dichlorophenol 6-monooxygenase is an oxidoreductase (monooxygenase). Transforms 2,4-dichlorophenol (2,4-DCP) into 3,5-dichlorocatechol, using NADH or NADPH as one donor and incorporating an atom of oxygen into the other donor.	[60]
	haloalkane dehalogenase	1-haloalkane + H_2_O = a primary alcohol + halide	Haloalkane dehalogenase is a hydrolase, has a broad substrate specificity, and catalyzes hydrolytic cleavage of carbon–halogen bonds in halogenated aliphatic compounds. Acts on a wide range of 1-haloalkanes, haloalcohols, haloalkenes, and some haloaromatic compounds, leading to the formation of the corresponding primary alcohols, halide ions, and protons.	[61,62,63]
	haloacetate dehalogenase	a haloacetate + H_2_O = a halide anion + glycolate + H^+^	Haloacetate dehalogenase is a hydrolase and catalyzes hydrolytic cleavage of carbon–halogen bonds in fluoroacetate (haloacetate dehalogenase DehH1), and chloro-, bromo-, and iodoacetate, but not fluoroacetate (haloacetate dehalogenase DehH2), yielding halide and glycolate.	[13,64,65,66]
	dichloromethane dehalogenase	dichloromethane + H_2_O = 2 chloride + formaldehyde + 2 H^+^	Dichloromethane dehalogenase is a lyase enzyme that generates formaldehyde and belongs to the family of glutathione S-transferases. Glutathione is required for its activity.	[67,68]
	alpha-ketoglutarate-dependent 2,4-dichlorophenoxyacetate dioxygenase	2,4-dichlorophenoxyacetate + 2-oxoglutarate + O_2_ = 2,4-dichlorophenol + CO_2_ + glyoxylate + succinate	Alpha-ketoglutarate-dependent 2,4-dichlorophenoxyacetate dioxygenase is an oxidoreductase (dioxygenase). Involved in degradation of the herbicide 2,4-dichlorophenoxyacetic acid (2,4-D). Is also able to degrade 2-methyl-4-chlorophenoxyacetic acid and 3-chlorobenzoic acid.	[69,70]
	*cis*-3-chloroacrylic acid dehalogenase		*Cis*-3-chloroacrylic acid dehalogenase is a hydrolase, which is involved in the hydrolytic cleavage of the vinylic carbon–halogen bond in 3-haloacrylates to afford malonate semialdehyde.	[71]
	tetrachlorohydroquinone reductive dehalogenase	2,6-dichlorohydroquinone + chloride + glutathione disulfide + H^+^ = 2,3,6-trichlorohydroquinone + 2 glutathione	Tetrachlorohydroquinone reductive dehalogenase is an oxidoreductase and is responsible for sequential reduction of tetrachloro-*p*-hydroquinone to monochlorophenol, using glutathione as the reducing agent.	[72]
	tetrachloroethene reductive dehalogenase	AH_2_ + tetrachloroethene = A + chloride + H^+^ + trichloroethene	Tetrachloroethene reductive dehalogenase is an oxidoreductase and a member of the reductive dehalogenase enzyme family. Catalyzes the reductive dechlorination of tetrachloroethene (PCE) to trichloroethene (TCE) and of trichloroethene to *cis*-1,2-dichloroethene (DCE). Can also use various chlorinated ethanes such as tetrachloroethane, pentachloroethane, and hexachloroethane. A—hydrogen acceptor; AH_2_—hydrogen donor.	[73]
**Cometabolic dehalogenases**	phenol monooxygenase	phenol + NADPH + H^+^ + O_2_ = catechol + NADP^+^ + H_2_O	Phenol monooxygenase has a broad substrate specificity and is an oxidoreductase (monooxygenase). It hydroxylates a variety of substituted phenols, such as fluoro- and chloro-phenols.	[13,43]
	toluene monooxygenase	H^+^ + NADH + O_2_ + toluene = 4-methylphenol + H_2_O + NAD^+^	Toluene monooxygenase is an oxidoreductase (monooxygenase) and uses the reducing power of NADH to split dioxygen and incorporate a single oxygen atom in the form of a hydroxyl group into the halogenated substrate, creating an unstable halohydrin. Subsequently, the unstable halohydrin spontaneously eliminates a halide ion.	[13,14,43]
	catechol 2,3-dioxygenase	catechol + O_2_ = 2-hydroxy-6-oxohexa-2,4-dienoate + H^+^	Catechol 2,3-dioxygenase belongs to the extradiol dioxygenase family and oxidoreductase class, and catalyzes ring cleavage of catechol and chloro-, methyl-, and ethyl-substituted catechols in a meta fashion.	[47,48,74,75,76]

## Data Availability

Research data are available upon request to the authors.

## Supplementary Materials

The following supporting information can be downloaded at: https://www.mdpi.com/article/10.3390/microorganisms11071702/s1, Table S1: Accession numbers of the detected dehalogenases; Table S2: Organochlorine pesticides content in soil and sediment samples obtained using a gas chromatograph coupled with an electron capture detector (GC-ECD). LOR—limit of reporting.
